# Myocardial Extracellular Volume Quantification by Cardiovascular Magnetic Resonance and Computed Tomography

**DOI:** 10.1007/s11886-018-0961-3

**Published:** 2018-03-06

**Authors:** Paul R. Scully, Gorka Bastarrika, James C. Moon, Thomas A. Treibel

**Affiliations:** 1 Cardiac Imaging Department, Barts Heart Centre, St Bartholomew’s Hospital, 2nd Floor, King George V Building, West Smithfield, London, EC1A 7BE UK; 2 Institute of Cardiovascular Science, University College London, Gower Street, London, WC1E 6BT UK; 3 Clínica Universidad de Navarra, University of Navarra, Avda/Pio XII 55, 31008 Pamplona, Spain

**Keywords:** Extracellular volume, Computed tomography, Cardiovascular magnetic resonance, Tissue characterization

## Abstract

**Purpose of review:**

This review article discusses the evolution of extracellular volume (ECV) quantification using both cardiovascular magnetic resonance (CMR) and computed tomography (CT).

**Recent findings:**

Visualizing diffuse myocardial fibrosis is challenging and until recently, was restricted to the domain of the pathologist. CMR and CT both use extravascular, extracellular contrast agents, permitting ECV measurement. The evidence base around ECV quantification by CMR is growing rapidly and just starting in CT. In conditions with high ECV (amyloid, oedema and fibrosis), this technique is already being used clinically and as a surrogate endpoint. Non-invasive diffuse fibrosis quantification is also generating new biological insights into key cardiac diseases.

**Summary:**

CMR and CT can estimate ECV and in turn diffuse myocardial fibrosis, obviating the need for invasive endomyocardial biopsy. CT is an attractive alternative to CMR particularly in those individuals with contraindications to the latter. Further studies are needed, particularly in CT.

## Introduction

Myocardial fibrosis is a frequently unwanted, common end point in the majority of pathological mechanisms affecting heart muscle. It can occur as focal scarring due to replacement fibrosis following myocyte death (apoptosis, autophagy or necrosis) or as diffuse fibrosis due to expansion of the collagen fibre network around individual myocytes or myocyte bundles [1]. The best non-invasive technique for visualising focal fibrosis is cardiovascular magnetic resonance (CMR) using late gadolinium enhancement (LGE), because of the high contrast, high spatial resolution and whole heart coverage. Although resultant image quality is currently reduced, myocardial fibrosis can also be assessed with cardiac computed tomography (CT)—both use an extracellular, extravascular contrast agent that lingers in extracellular water in areas of scar, due to a higher volume of distribution and slower kinetics. Visualization of diffuse fibrosis until now has remained challenging and limited to the domain of the pathologist, who was able to measure the extracellular matrix (ECM) directly on histological sections using stains specific for connective tissue [2]. Coupled with this, no useful blood biomarkers of myocardial fibrosis are currently extant. In the last 7 years, the same contrast agents have begun to be used to measure diffuse interstitial expansion (as well as focal scar), by measuring the extracellular volume (ECV). This review will focus on the quantification of ECV using the two most commonly used contrast agents: gadolinium and iodine based.

## Development of an Extracellular Contrast Agent

An extracellular contrast agent has a key set of properties: (1) homogeneous distribution; (2) high water, but no fat solubility; (3) not adsorbed, actively transported, protein-bound or metabolized; (4) non-toxic, stable and freely cleared from the body and (5) readily measurable. Iodine and gadolinium compounds both fulfil these requirements as contrast agents. They diffuse rapidly and passively from the vascular space into extracellular tissue, but not into the intracellular space—leading to the term ‘extracellular, extravascular contrast agent’. Following an intravenous bolus, they enter the myocardium down a concentration gradient (‘wash-in phase’), and later, while being cleared, they return to the blood pool down the reverse concentration gradient (‘wash-out phase’). This occurs over seconds to minutes in healthy myocardium, but in scar tissue (focal or diffuse), these pharmacokinetics are delayed due to changes in coronary flow rates, capillary permeability, functional capillary density and the presence of a dense, hydrated collagen matrix [3]. In addition, the increased volume of extracellular water present in scar compared to normal myocardium means total accumulation is higher. The combined result is that, at a certain time ‘late’ after a bolus, there is more contrast agent in scar than in the blood or remote myocardium and measurable signal is therefore changed.

In CMR, gadolinium-based contrast agents (GBCAs) are used due to their unique magnetic properties (gadolinium is a paramagnetic metal with the most unpaired electrons) [4]. They are particularly efficient T1-relaxing agents, resulting in increased signal on T1-weighted images and typically appearing bright on a T1 inversion recovery image. The relaxation rate (R1 or 1/T1) is directly proportional to the concentration of gadolinium. In CT, non-ionic iodinated contrast agents have become the most commonly used contrast agents [4, 5]; they are water-soluble, extracellular, extravascular contrast agents, which are not metabolized and are excreted by the kidneys [6, 7]. CT attenuation values (represented as Hounsfield units, HU) are directly proportional to the concentration of iodine.

## Extracellular Volume Imaging by CMR

Until recently, the gold standard of diagnosing diffuse fibrosis was endomyocardial biopsy, which is invasive (carrying risk) and is prone to sampling errors. This has led to the development of new, non-invasive techniques to better quantify diffuse fibrosis. CMR allows non-invasive tissue characterization of the myocardium and as such, is being increasingly used to identify the aetiology of a range of cardiomyopathies. LGE is the mainstay of this myocardial characterization and allows the detection of focal fibrosis [3, 8]. This technique combined with functional imaging is the main reason that CMR is so useful clinically. LGE imaging relies on the delayed post-contrast difference in T1 between areas of fibrosis (more gadolinium, shorter T1) and healthy myocardium (less gadolinium, longer T1) [9], making it ideal for identifying focal areas of fibrosis. In diffuse fibrosis, this relative difference is lost, so conventional LGE imaging struggles—being a difference test where the operator selects one ‘normal’ tissue to null making all other tissues ‘bright’[9, 10••]. GBCAs change tissue T1; however, the native (non-contrast) T1 also changes with pathology. Advances in CMR sequences now permit its quantification via T1 mapping, which offers absolute values of T1, rather than relative differences in signal intensity. [10••]

Native T1 describes the signal in the whole of the measured myocardium and therefore represents a composite signal from all species present—this signal is swamped by iron or gadolinium if present and in their absence is measuring the signal of both the cardiac myocytes and the ECM [10••]. Therefore, fibrosis/oedema/amyloid and associated water increase T1, and conversely increased cellularity (athleticism), iron (thalassemia) or fat (Anderson Fabry disease) decrease T1 [10••, 11•, 12•].

The use of extracellular GBCAs in CMR offers the opportunity to quantify the extracellular (i.e. interstitial) space, relative to the intracellular (i.e. myocyte) space, which is the essence of ECV quantification. It dichotomises the myocardium into myocytes and matrix. In conjunction with myocardial volume, ECV can be used to calculate the relative volumes of each compartment. It is expressed as a volume fraction and provides us with unique insights into the pathophysiology of a range of myocardial diseases [10••].

T1 mapping and ECV may provide an advantage over conventional LGE imaging, by enabling us to more accurately quantify diffuse fibrosis and potentially detect early fibrosis-related changes not always detectable by LGE [13]. Indeed, increases in ECV seen on CMR are associated with an increased mortality and may be as important to prognosis as left ventricular ejection fraction [13, 14••]. In-depth discussion of the advantages and limitations of native T1 mapping are beyond the scope of this review and have been described elsewhere [8, 11•]; instead, this review will focus on ECV imaging, which combines pre- and post-contrast images.

## Evolution of ECV by CMR

Initial validation in humans utilizing CMR to quantify ECV (ECV_CMR_) and in turn diffuse myocardial fibrosis was performed in 2010 by Flett et al. [15]. They employed a technique they termed ‘equilibrium contrast CMR’, which involved an initial bolus of GBCA, followed by a continuous infusion to achieve an equilibrium of contrast between the blood pool and the myocardium [15]. They estimated the blood volume of distribution from 1-haematocrit and then used CMR to measure the pre- and post-contrast equilibrium T1 [15]. Using the formula below, they then calculated the ECV.

$$ {\mathrm{ECV}}_{\mathrm{CMR}}=\left(1\hbox{--} \mathrm{haematocrit}\right)\times \left(\Delta \left(1/{\mathrm{T}1}_{\mathrm{myo}}\right)/\Delta \left(1/{\mathrm{T}1}_{\mathrm{blood}}\right)\right) $$

They validated this technique by direct comparison with histological fibrosis quantification using picrosirius red staining on surgical biopsy samples from patients undergoing aortic valve replacement for aortic stenosis (AS, *n* = 18) and myomectomy for hypertrophic cardiomyopathy (HCM, *n* = 8) and showed excellent correlation (combined *r*^2^ = 0.80) [15]. Similar studies correlating ECV_CMR_ with histology have been reproduced in patients with aortic and mitral regurgitation [16], dilated cardiomyopathy (DCM) or ischaemic heart disease (IHD) awaiting cardiac transplantation [17], heart failure [18] and myocarditis [19]. See Table 1 for more details.

**Table 1:** Histological validation of ECV by modality

Reference	Year	Population	Number	Finding
ECV by CMR				
Flett et al. [15]	2010	AS/HCM	26	Strong correlation with histological fibrosis in AS (*r*^2^ = 0.86) and HCM (*r*^2^ = 0.62).
White et al. [21]	2013	AS	18	Bolus only and infusion ECV measurements correlated with histological CVF (*r*^2^ = 0.69 and 0.71).
Miller et al. [17]	2013	DCM/IHD (transplant)	6	Significant linear relationship with histological CVF using either the 10- or 15-min post-contrast T1 (*p* < 0.001).
De Meester et al. [16]	2015	AS/AR/MR	31	Strong correlation with the magnitude of histological fibrosis (*r* = 0.78).
Kammerlander et al. [18]	2016	Mixed HF	36	Significant correlation with histological CVF (*r* = 0.493).
Lurz et al. [19]	2016	Myocarditis	129	ECV adequately estimated the degree of LV fibrosis percentage only in patients without inflammation (r = 0.72) and not in those with inflammation (*r* = 0.24).
ECV by CT				
Bandula et al. [34]	2013	AS	23	Strong correlation with histological measures of fibrosis (*r* = 0.71).
Yoon et al. [43]	2015	Hepatic fibrosis	141	Significant correlation with histological hepatic fibrosis staging (*r* = 0.493).

*AS* aortic stenosis, *AR* aortic regurgitation, *CVF* collagen volume fraction, *DCM* dilated cardiomyopathy, *HCM* hypertrophic cardiomyopathy, *HF* heart failure, *IHD* ischemic heart disease, *MR* mitral regurgitation

A significant barrier to the adoption of the ECV_CMR_ technique was the use of the primed infusion protocol. This involved the patient being removed from the scanner after conventional LGE imaging and being given another bolus of GBCA, followed by a 15-min pause and then an infusion. The patient would then be returned to the scanner any time between 45 and 80 min after the bolus for repeat T1 measurement [15].

A bolus-only approach was proposed by Schelbert et al., who demonstrated in 10 volunteers that myocardial ECV_CMR_ could be reliably measured 15–20 min after a single bolus of GBCA [20]. Further work by White et al., this time in 147 subjects, demonstrated a strong correlation between 15-min bolus only and infusion ECV_CMR_ measurement (*r*^2^ = 0.97) [21]. They did note that when the ECV was > 40%, the bolus only technique consistently measured a higher ECV than the infusion [21]. Finally, the validation of ECV_CMR_ as part of a split-dose protocol (e.g. as part of stress perfusion) by McDiarmid et al. further increased the potential clinical utility of the technique [22]; however, there are suggestions that ECV_CMR_ values may differ depending on the dose of GBCA used [23].

Most recently, a synthetic ECV can be automatically generated during scanning, in which the haematocrit of blood is inferred from the T1 of the blood pool (as the relationship between haematocrit and R1 [1/T1_Blood_] is linear), removing the need for a blood test [24]. It has also been replicated at 1.5 and 3T on other scanner platforms [25]. The key advantage of this technique is the simplification of the ECV workflow—by removing the need for blood tests to measure haematocrit, which is burdensome in busy departments, is a source of user error and introduces reporting delay. Implementation of inline synthetic ECV tools (with instantaneous ECV maps) would reduce the barriers to clinical use of ECV and potentially increase quality of care as review is immediately available.

## ECV by CMR in Clinical Practice

Normal ECV values depend on the field strength, T1 mapping sequence and scanner manufacturer, but range between 20 and 26%, and appear to be slightly higher in women compared to men [26]. With the exception of cardiac amyloidosis and oedema [27], increases in myocardial ECV are generally due to an increased collagen volume fraction (CVF)—making it a marker of fibrosis [8, 10••]. For example, acute myocardial infarction (MI) results in some of the highest ECV values (58.5 ± 7.6%) and chronic MI is not far behind (51 ± 8%) [26, 28]. Diffuse fibrosis, however, rarely increases beyond 40%. Cardiac amyloidosis, which is characterized by the *extracellular* deposition of misfolded protein, produces large increases in ECV (greater than any other non-ischemic cardiomyopathy) in the region of 46.6 ± 7% [26, 27, 29].

**Fig. 1:**
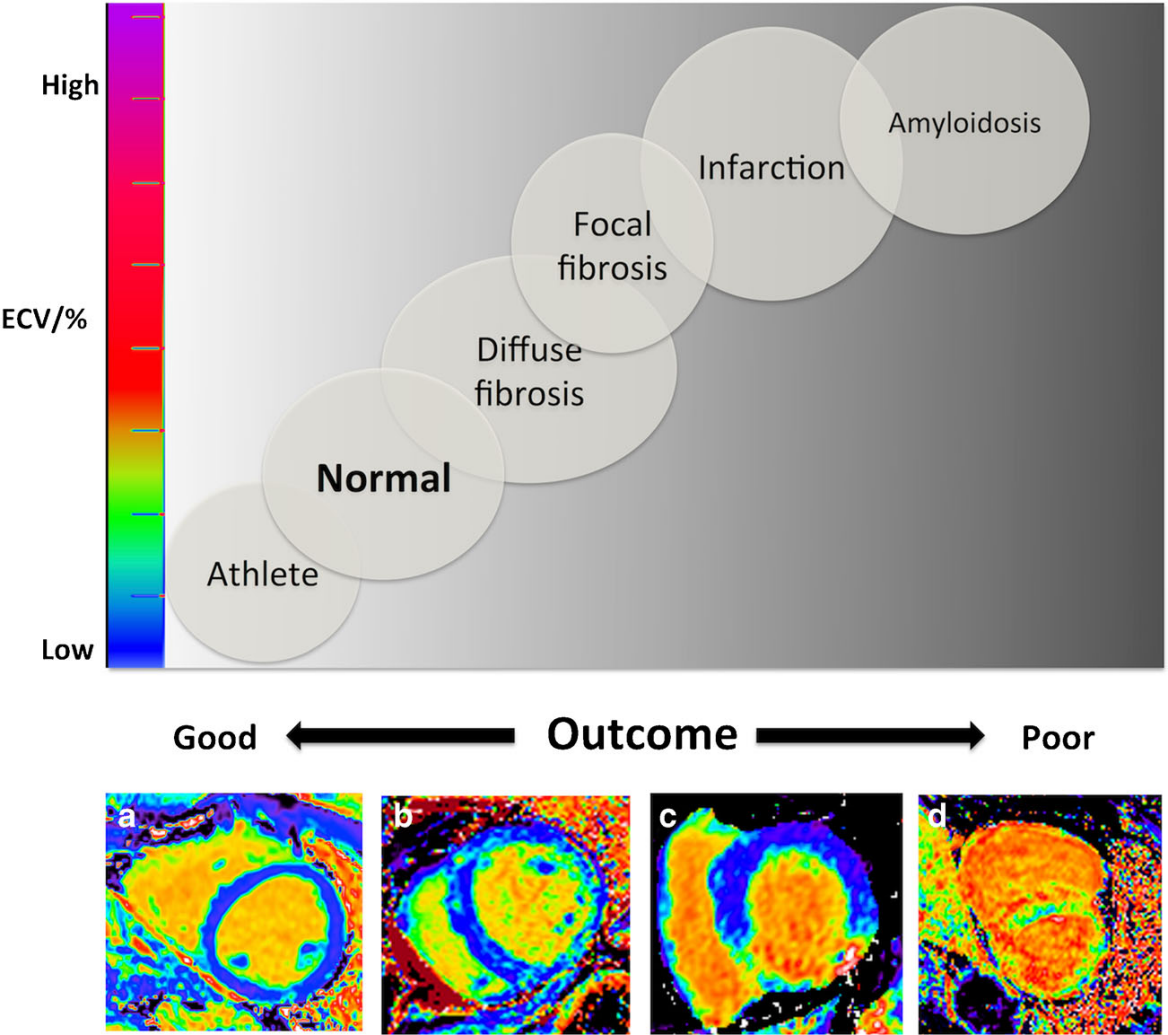
Extracellular volume fraction (ECV) variability and outcome at 1.5T by myocardial pathologies. Top panel depicts ECV and associated outcome across health and disease with increasing ECV on the *y*-axis and outcome on the *x*-axis. Bottom panel shows four exemplar ECV maps of a healthy volunteer with normal ECV of 24% (**a**), a patient with aortic stenosis with mild ECV elevation at 30% (**b**), a patient with an inferior myocardial infarct (**c**), and a patient with AL cardiac amyloidosis with an ECV of 50% and the poorest outcome (**d**). (Adapted from Ugander 2014) [86]

ECV is also mildly elevated in both hypertrophic cardiomyopathy (29.1 ± 0.5%) and dilated cardiomyopathy (28 ± 0.4%) [26]. On the other hand, Anderson Fabry disease appears to have a similar ECV to healthy volunteers (25.0 ± 2.3%), at least in the early stages of the disease [8, 26]. For an overview of ECV variability in health and disease, see Fig. 1 and Table 2.

**Table 2:** Overview of ECV imaging in myocardial disease

Process	Disease	Number of patients	ECV
Athletic hypertrophy			
	Physiological hypertrophy [12•]	30	**↓**
Fibrosis			
Focal	Myocardial infarction [26, 28]	56	**↑↑↑**
Diffuse	Aortic stenosis [26, 61, 62]	136	**— /↑**
	Systolic heart failure [63]	40	**↑**
	Diastolic heart failure [63]	62	**↑**
	Hypertrophic cardiomyopathy [64, 65]	102	**↑**
	Non-ischemic dilated cardiomyopathy [65, 66]	116	**↑**
	Mitochondrial cardiomyopathy [67]	1	**↑**
	Diabetes [14••]	231	**↑**
	Hypertensive heart disease [68, 69]	89	**–**
	Obesity [70]	21	**↑**
	Congenital heart disease [71]	14	**↑**
Inflammation			
	Rheumatoid arthritis [72]	39	**↑↑**
	Systemic sclerosis [73, 74]	49	**↑↑**
	Systemic lupus erythematosus [75]	33	**↑↑**
Oedema			
Regional	Myocarditis [76, 77]	135	**↑**
Global	Anti-synthetase syndrome [78]	1	**↑↑**
	Chronic systemic capillary leak syndrome [79]	6	**↑↑**
	Acute cardiac allograft rejection [80]	22	**–**
Infiltration			
Amyloid	AL amyloid [81]	100	**↑↑↑**
	TTR amyloidosis [82]	102	**↑↑↑**
Glycosphingolipid	Anderson-Fabry disease [83]	31	**–**
Toxins			
	Uraemia in chronic kidney disease [84]	43	**↑**
	Anthracycline-toxicity [85]	30	**— /↑**

Reference list is non-exhaustive—several other references may exist that are not listed here

— No significant change; *↑* Significant increase; ↓ Significant decrease.

(Adapted from Captur et al. Heart 2016, Sep 15;102 (18):1429–35, with permission from BMJ Publishing Group Ltd.) [87].

## Evolution of ECV by CT

ECV imaging by cardiac CT (ECV_CT_) lags behind the CMR field, but is potentially an attractive alternative. ECV_CT_ relies upon the same principle as ECV_CMR_ and is calculated using the following formula:

$$ {\mathrm{ECV}}_{\mathrm{CT}}=\left(1\hbox{--} \mathrm{haematocrit}\right)\times \left({\Delta \mathrm{HU}}_{\mathrm{myo}}/{\Delta \mathrm{HU}}_{\mathrm{blood}}\right) $$

where ΔHU is the change in Hounsfield unit attenuation pre- and post-contrast (i.e. HU_post-contrast_ − HU_pre-contrast_) [30–32].

Early data was presented in abstract form by Ugander et al. in 2011, which showed, in dogs that underwent coronary occlusion and reperfusion (*n* = 10), that ECV_CT_ and ECV_CMR_ correlated well (*R*^2^ = 0.80, *p* < 0.001), with a small mean difference between ECV_CMR_ and ECV_CT_ (3 ± 9%) [33]. Myocardial ECV_CT_ was first validated in humans by Nacif et al. in 2012 [30]. They compared ECV_CMR_ and ECV_CT_ in 24 subjects (both healthy volunteers and those with heart failure) and found good correlation between the two (*r* = 0.82, *p* < 0.001) [30]. Post-contrast images were taken after a 10-min delay, copying the exact parameters of the initial pre-contrast calcium score scan. Overall radiation dose was low (< 2 mSv) [30]. The average duration of ECV_CT_ in this study was 13 ± 1.5 min, compared to 47 ± 5 min for ECV_CMR_ [30]. ECV_CMR_ results were slightly lower (28.6 ± 4.4%) compared to ECV_CT_ (31.6 ± 5.1%) [30]. The same group went on to compare ECV_CT_ in healthy volunteers and those with either systolic or diastolic heart failure [31]. They used a similar protocol, only this time, taking the post-contrast images after a shorter 7-min delay [31]. They found that the ECV was significantly higher in participants with systolic heart failure (41 ± 6%) compared to healthy subjects (33 ± 2%) and those with diastolic heart failure (35 ± 5%) [31].

Bandula et al. in the same year validated ECV_CT_ against the gold standard—invasive endomyocardial biopsy, as well as ECV_CMR_ in 23 patients with severe aortic stenosis [34]. This time, they used an initial bolus of contrast agent, followed by a slow infusion to achieve equilibrium. They found that ECV_CT_ showed significant correlation with both histological measures of fibrosis (*r* = 0.71, *p* < 0.001) and ECV_CMR_ (*r* = 0.73) [34]. We subsequently compared 5- and 15-minute time points post-contrast bolus for equilibrium cardiac CT in 53 patients (26 with systemic amyloidosis and 27 with aortic stenosis) [32]. We demonstrated that ECV_CT_ at 5-minute post-contrast showed a stronger correlation with ECV_CMR_ than at 15-minute post-contrast (*r*^2^ = 0.85 compared to 0.74) [32]. We think this is because, although earlier imaging risks non-equilibration, iodine is a weaker contrast agent than GBCAs, so the higher signal-to-noise ratio of earlier imaging outweighs this. ECV_CT_ was consistently higher in those patients with confirmed cardiac amyloid compared to those with aortic stenosis (54 ± 11% compared to 28 ± 4%, *p* < 0.001) and was able to discriminate between patients with definite cardiac amyloid and those with aortic stenosis in all cases [32]. ECV_CT_ also tracked various important clinical parameters including reduced 6-min walk test distance and increasing NT-proBNP, as well as amyloid burden in transthyretin-related amyloid—when measured semi-quantitatively by 99mTc-3,3-diphsphono-1,2-propanodicarboxylic acid (DPD) scintigraphy [32].

Work is also being done using dual-energy CT to quantify myocardial ECV, which is an attractive concept, as it would potentially avoid mis-registration errors associated with the separate pre- and post-contrast scans, by obviating the need for the pre-contrast scan [35, 36]. Hong et al. used dual-energy CT to estimate myocardial ECV in doxorubicin-induced dilated cardiomyopathy in rabbits [36]. They showed equivalent ECV results by dual-energy CT from 3 up to 20 minute post-contrast administration [36]. ECV values were significantly higher at 6, 12 and 16 weeks after starting twice weekly doxorubicin injections than at baseline (35.3, 41.9, 42.1% vs. 28.5%) [36]. ECV measured by dual-energy CT showed excellent correlation with ECV_CMR_ (*r* = 0.888, *p* < 0.001) and with collagen volume fraction on histology (*r* = 0.925, *p* < 0.001) [36]. Work has also been done in humans using dual energy CT—involving 30 subjects (7 healthy, 23 with hypertrophic or dilated cardiomyopathy, amyloidosis or sarcoidosis) [37]. The post-contrast dual-energy CT scan was performed at 12 minute and results for ECV were compared with CMR_ECV_ and showed good agreement. Those participants with disease had significantly higher myocardial ECV by dual-energy CT compared to healthy subjects (*p* < 0.01) [37].

More recently, synthetic ECV has also been successfully implemented in CT, where the haematocrit of blood is inferred from the attenuation of the blood pool (as the relationship between haematocrit and HU is also linear), simplifying the ECV workflow and allowing instantaneous display of ECV maps [38].

## CT versus CMR for Myocardial ECV Quantification

While the evidence base for ECV_CMR_ may be larger and experience greater, the use of CT in this regard does have some distinct advantages. Likely to prove the biggest advantage is that ECV_CT_ measures the direct effect of iodine-based contrast agents on the measured signal (through the effect of iodine on x-ray absorption), whereas ECV_CMR_ relies on measuring the effect of GBCAs on protons (therefore making two assumptions—the first is that the relaxivity of tissues compared to blood is the same and the second is that water is rapidly exchanged between intra- and extracellular compartments). Also, common contraindications to CMR such as claustrophobia and pacemaker implantation (the latter being not uncommon in patients with for example cardiac amyloid—where ECV quantification could prove helpful) do *not* apply for CT.

ECV_CMR_ is likely to be costly, both financially and in terms of patient throughput—with scans taking up to 60 min, whereas CT scans are significantly faster and more widely available. Indeed, CT availability is only likely to increase, particularly in the UK, given the expanded role for CT coronary angiography in the 2016 update of the National Institute for Health and Care Excllence clinical guideline (CG95) on the assessment of chest pain of recent onset [39]. CT also offers higher spatial resolution particularly inplane and allows isotropic reconstruction. Of course, these potential advantages should be weighed up against the risks of ionizing radiation from CT, especially in younger patients. Furthermore, CT is also prone to artefacts, e.g. beam hardening and streak artefacts that may hamper the evaluation of myocardial ECV. Finally, in delayed acquisitions, iodine contrast media provide less signal compared to GBCAs and differentiation between myocardium and left ventricular cavity is hampered, particularly when the myocardium is thinned (for example in dilated cardiomyopathy).

GBCAs have been associated with the development of nephrogenic systemic fibrosis, seen with linear chelates rather than macrocyclic and in patients with significantly impaired renal function (eGFR < 30 mL/min/1.73 m^2^) [40]. These linear agents have also been associated with brain deposition, a currently evolving story (but apparently not seen with macrocyclic chelates [41]. Iodinated contrast agents have in turn been associated with contrast-induced nephropathy and pre-existing chronic kidney disease (eGFR < 60 mL/min/1.73 m^2^) is the most important risk factor for this [42].

It is important to bear in mind while considering the pros and cons of both of these modalities that relative to the current gold standard for diagnosing diffuse fibrosis of invasive endomyocardial biopsy, both offer very attractive alternatives.

## ECV_CT_ in Clinical Practice—from Research Tool to Clinical Application

For clinical utilization, there needs to be standardized protocols in place for performing ECV_CT_. Furthermore, we need to employ this technique to better diagnose and understand disease processes and the effect of treatment on ECV (for example as a surrogate end point in drug trials). This has been implemented in the T1 mapping consensus statement, with a second version to be published in mid-2017 [10••]. For ECV_CT_ to become a technique used in clinical practice, key steps need to be implemented—similar to developments in the CMR community, in particular standardization with phantom work, multicentre clinical data in health and disease, a consensus statement by national/international organizations (e.g. EACVI/SCCT) and adoption by all major manufacturers.

## ECV Quantification beyond the Myocardium

The potential for ECV_CT_ and ECV_CMR_ extends beyond the myocardium. Yoon et al. used CT to estimate liver ECV in order to measure hepatic fibrosis and validated this against histological hepatic fibrosis staging in 141 participants (*r* = 0.493, *p* < 0.001) [43]. Bandula et al. also demonstrated elevated ECV_CMR_ in the liver, spleen and skeletal muscle in patients with systemic amyloidosis, which tracked semi-quantitative amyloid burden in the liver and spleen by serum amyloid P component (SAP) scintigraphy [44]. Similar work has been performed by Yeung et al. using ECV_CT_ [45]. They showed that in patients with hepatic and splenic amyloid, there was a significantly higher ECV compared to patients without liver and spleen involvement (*p* < 0.0005) [45]. They were also able to show that increases in ECV_CT_ positively correlated with the grade of hepatic and splenic uptake on SAP scintigraphy (*r* = 0.758 for liver, *r* = 0.867 for spleen) [45].

## Future Outlook for CT

Currently, a poorer signal-to-noise ratio than CMR and the use of ionizing radiation have impeded wider application of myocardial tissue characterization by CT. Nevertheless, the possibility of providing an assessment of coronary anatomy, coronary flow and myocardial tissue characterization in a single modality is an attractive concept, with huge implications for imaging workflow.

Beyond the optimization of dual-energy CT with minimization of image artefacts, radiation dose and iodinated contrast dose (e.g. using low-energy monochromatic imaging [46]), more advanced technologies are on the horizon. Spectral CT imaging exploits the different K-edge behaviour of different tissues (calcium, blood, fat and myocardium) [47]. This technology goes beyond the two-photon energy levels used in dual-energy CT, and utilizes energy-sensitive photon-counting detectors to obtain greater tissue information by differentiating photons at different energy levels. Early pre-clinical data suggests that spectral CT may improve image quality over conventional CT by eliminating beam hardening [48].

## Future Outlook for CMR

The T1 mapping field has been rapidly advancing to the point of widespread clinical utility. Since the first T1 quantification with the original modified look-locker imaging (MOLLI) in 2004 [49], new MOLLI variants, ShMOLLI (a shortened variation with long T1 advantages) [50], saturation recovery variants such as SASHA (offering complete heart rate insensitivity) [51] or hybrid approaches [52–54] have been developed, and incremental developments such as respiratory motion correction [55] have gradually increased accuracy and precision [52, 56•]. ECV maps are now routine in some centres [57], but ECV development and standardization are still on-going and will require global approaches. Quality control systems, commercial sequences, mega-registries (e.g. the Global CMR Registry, HCM Registry and UK Biobank) are in progress, and will provide high volumes and new insights into the currently most active CMR research area [58, 59]. On the horizon, MR fingerprinting may offer more rapid multi-parametric tissue characterization in the future by providing myocardial T1, T2, and Proton Spin Density in a single breath-hold [60].

## Conclusion

Myocardial ECV is important, with increases related to myocardial fibrosis, cardiac amyloid or oedema, which in turn are associated with an increased mortality. Quantification of ECV enables the detection of diffuse myocardial fibrosis, which would otherwise potentially be missed using conventional LGE imaging, making it a useful addition to the armamentarium of myocardial characterization techniques.

CT and CMR can be used to estimate myocardial ECV and in turn diffuse myocardial fibrosis, without the need for invasive endomyocardial biopsy. Each modality has strengths and weaknesses, with CT an attractive alternative to CMR particularly in those with contraindications to the latter. Further studies are needed in this field, especially ECV_CT_, where the evidence base is less robust.

## Abbreviations

AS: Aortic stenosis
AR: Aortic regurgitation
CMR: Cardiovascular magnetic resonance
CT: Computed tomography
CVF: Collagen volume fraction
DCM: Dilated cardiomyopathy
ECM: Extracellular matrix
ECV: Extracellular volume
ECV_CMR_: Extracellular volume quantified by cardiovascular magnetic resonance
ECV_CT_: Extracellular volume quantified by computed tomography
HCM: Hypertrophic cardiomyopathy
HF: Heart Failure
GBCAs: Gadolinium based contrast agents
IHD: Ischaemic heart disease
LGE: Late gadolinium enhancement
MI: Myocardial infarction
MR: Mitral regurgitation
SAP: Serum amyloid P component
